# Efficacy of tenofovir in preventing perinatal transmission of HBV infection in pregnant women with high viral loads

**DOI:** 10.1038/s41598-018-33833-w

**Published:** 2018-10-19

**Authors:** Yayun Lin, Yan Liu, Guifeng Ding, Lhousseine Touqui, Weimin Wang, Na Xu, Keying Liu, Lingyan Zhang, Dunjin Chen, Yongzheng Wu, Guiqin Bai

**Affiliations:** 1 Department of Gynecology and Obstetrics, The First Affiliated Hospital of Xi’an Jiaotong University, Xi’an, China; 2 Research Center for Clinical and Translational Medicine/Institute of Infectious Diseases, Beijing 302 Hospital, Beijing, China; 3 Department of Obstetrics, Maternal and Child Health Care Hospital of Xinjiang Uygur Autonomous Region, Urumqi, China; 4 Equipe Mixte Institut Pasteur/Paris V, Department of Infection & Epidemiology, Institut Pasteur, Paris, France; 5 Department of Gynecology and Obstetrics, ShaanXi Provincial People Hospital, Xi’an, China; 6 Third Affiliated Hospital of Guangzhou Medical University, Guangzhou, China; 7 Unit of Cellular Biology of Microbial Infection/CNRS UMR3691, Institut Pasteur, Paris, France

## Abstract

Mother-to-child transmission is the major cause of chronic hepatitis B virus (HBV) infection. This double-blind trial tested the effect of tenofovir disoproxil fumarate (TDF) in preventing vertical transmission. Pregnant women who were HBsAg/HBeAg-positive with a HBV DNA titer ≥ 2×10^6^ IU/mL were randomly assigned to the control (n = 60) and TDF-treated (n = 60) groups. TDF treatment (oral dose 300 mg/day) was initiated at 24 weeks of gestation and continued to 4 weeks after delivery. The subjects were followed up to 28 weeks postpartum. The effects of TDF on vertical transmission, outcomes of the mothers and infants and virological changes were monitored. TDF dynamically reduced the serum HBV DNA level of the mothers, particularly during the first 4 weeks of treatment. The lower viral loads were maintained in the pregnancies until delivery. Approximately 90% and 33.9% of the TDF-treated mothers had viral loads ≤2000 IU/mL after delivery and at 28 weeks postpartum, respectively. No cervical transmission or adverse effects were observed in the TDF-treated individuals, whereas 13.5% of the infants were infected with HBV in the control group. We conclude that TDF treatment initiated at 24 weeks of gestation in high-viremia, HBsAg/HBeAg-positive mothers efficiently prevents mother-to-child HBV transmission without adverse events in mothers and infants.

## Introduction

Chronic hepatitis B virus (HBV) infection is a major public health concern. Approximately 350 million individuals worldwide are estimated to be HBV carriers, and 650,000 deaths each year are associated with HBV infection^1,2^. Asian populations have a much higher prevalence of HBV than other populations^3^. As an endemic region, China has approximately 130 million HBV carriers and 30 million chronic HBV infections^4^.

HBV can infect people through acceptance of blood products or drug injection^5^. However, due to the special immune status in pregnancies that allow mothers to tolerate the semiallogeneic fetus, mother-to-child transmission has become another major pathway for HBV infection, especially for mothers who are hepatitis B e antigen (HBeAg)- and hepatitis B surface antigen (HBsAg)-positive with high viremias^6,7^. Indeed, infants born to HBsAg-positive mothers (0.6 to 5.8% of pregnant women) have an increased risk for potential chronic HBV infection^8,9^. More than 90% of infants born from HBeAg-positive pregnancies will be infected with HBV if immunoprophylaxis is not accepted^10^. In addition, HBV-infected infants have a higher risk of developing chronic HBV infection than those at other ages^11^. Strikingly, mother-to-child transmission of HBV infection has decreased dramatically by 75–90% due to global immunoprophylaxis^1,2,12–14^. However, immunoprophylaxis still fails in 10–30% of infants, who develop chronic HBV infection through vertical transmission^15–17^. High viral burdens in mothers have been reported as the major factor underlying this prophylaxis failure and vertical transmission^2,10,18,19^. Furthermore, HBV-infected children also have higher potential for liver cirrhosis and hepatocellular carcinoma. Thus, prevention of mother-to-child transmission, such as through antiviral treatment during pregnancy, has become the key issue to achieve the global goal of eliminating HBV infection.

Currently, nucleotide analogues (NAs), including lamivudine, telbivudine and tenofovir disoproxil fumarate (TDF), are used to control HBV infection. As a nucleotide reverse transcriptase inhibitor, TDF is the only approved NA with high efficacy against the virus but no detected clinical resistance to date; thus, this drug has been widely used against HIV or HIV/HBV co-infection^20^. In 2015, TDF treatment was recommended by the WHO against chronic HBV infection, particularly in pregnant women^21^. However, as a result of limited efficacy and safety data, the FDA of the USA still categorizes TDF as a “B” class reagent. Indeed, controversial observations have been achieved for TDF application. For instance, application of TDF at a late stage of pregnancy reduced the serum HBV DNA level and vertical transmission of HBV^3,17,22–24^, but perinatal transmissions or adverse events were still reported in these trials; initiation of TDF treatment during the second trimester of gestation was also shown to block mother-to-child transmission but still had severe adverse effects^25–28^. Thus, evaluating the efficacy and safety of TDF treatment in pregnancies with HBV-infected mothers is critical.

This clinical trial was designed for pregnancies with HBsAg/HBeAg double-positive mothers with serum HBV DNA ≥ 2×10^6^ (6.3 log10) IU/mL. TDF therapy was initiated at 24 weeks of gestation and continued to 4 weeks after delivery. Dynamic changes in the viral loads, vertical transmission and maternal/fetal outcomes were evaluated.

## Results

**Figure 1:**
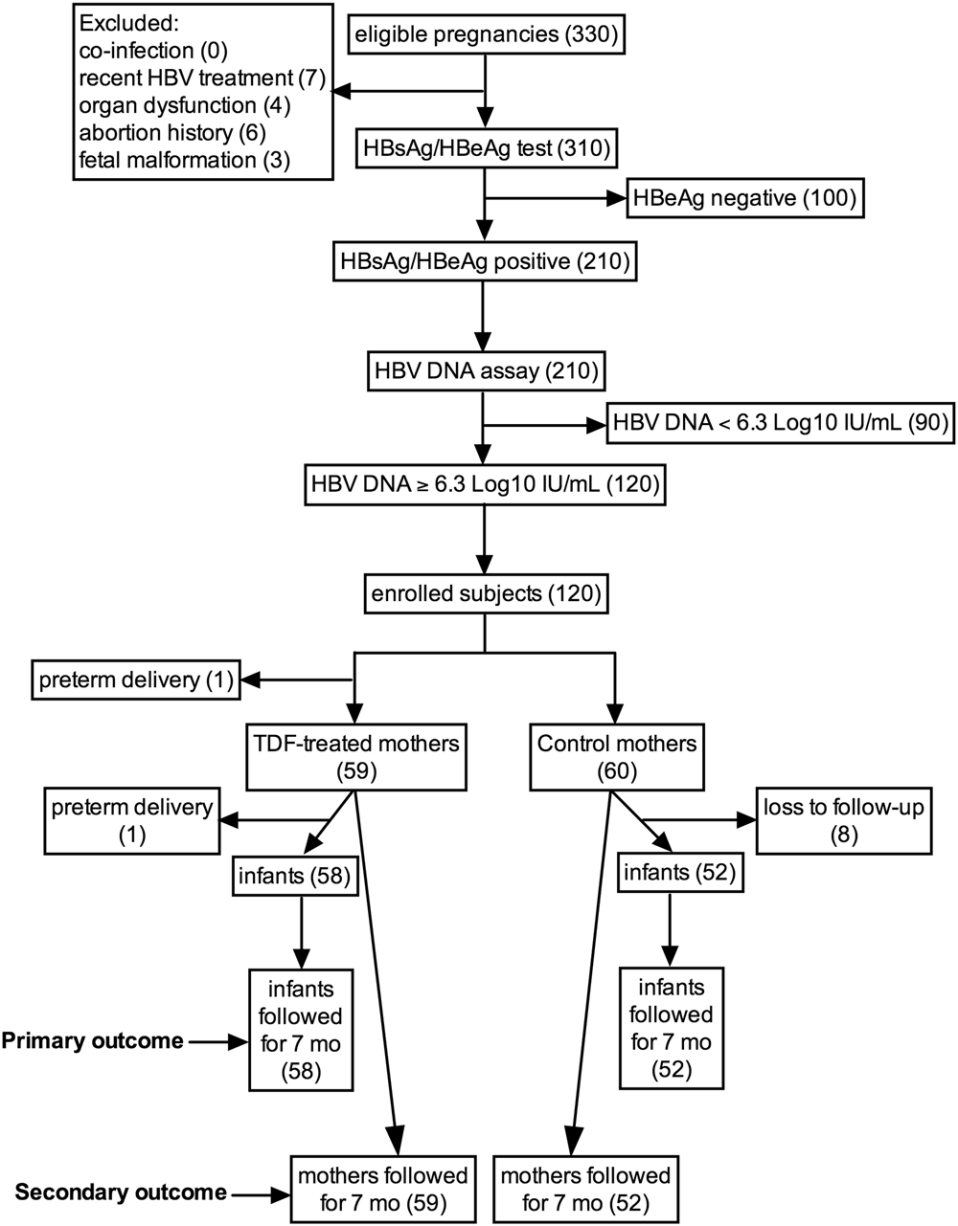
The subject selection flow chart.

As shown in the subject selection flow chart (Fig. 1), the pregnant women were randomly divided into two groups. In the TDF treatment group, one abortion occurred at 12 weeks of gestation, and the other 59 pregnancies accepted TDF therapy. Of these pregnancies, 1 case of premature delivery occurred at 32 weeks of gestation, and the premature infants died from hypoxic brain disease. In the control group, 8 subjects were lost to follow-up, and the other 52 pregnancies accepted routine nursing and prenatal care until the end of the study. In total, 110 pregnancies successfully participated in the whole trial process, and 110 infants were born. Table 1 shows the basic characteristics and clinical features of these pregnant women. No significant differences (p > 0.05) were observed between the TDF-treated and control groups in terms of age, pregnancy times, treatment periods, basal ALT, HBV genotype, occupation, native place, residence, education level and family income.

**Table 1:** The basic information and clinical features of the pregnant women.

		Groups				t	p value
		TDF treatment (n = 59) Mean ± SD	%	Control (n = 52) Mean ± SD	%		
Pregnant women	Age (y)	28.31 ± 3.56		28.06 ± 3.42		0.379	0.705
	Pregnancy number	1.28 ± 0.451		1.25 ± 0.437		0.305	0.761
	Basal ALT level (U/L)	54.62 ± 105.7		57.5 ± 103.3		−0.145	0.88
	HBV genotype						0.809
	B2	4	6.9	3	5.8		
	C2	54	93.1	49	94.2		
Family status	Domicile						0.262
	Rural	33	56.9	35	67.3		
	City	25	43.1	17	32.7		
	Residency						0.994
	Temporary	19	32.8	17	32.7		
	Resident	39	67.2	35	67.3		
	Education						0.823
	Below college	39	67.2	36	69.2		
	College and above	19	32.8	16	30.8		
	Family incomes (year)						0.976
	<40,200 RMB	18	31.0	16	30.8		
	>40,200 RMB	40	69.0	36	69.2		

**Figure 2:**
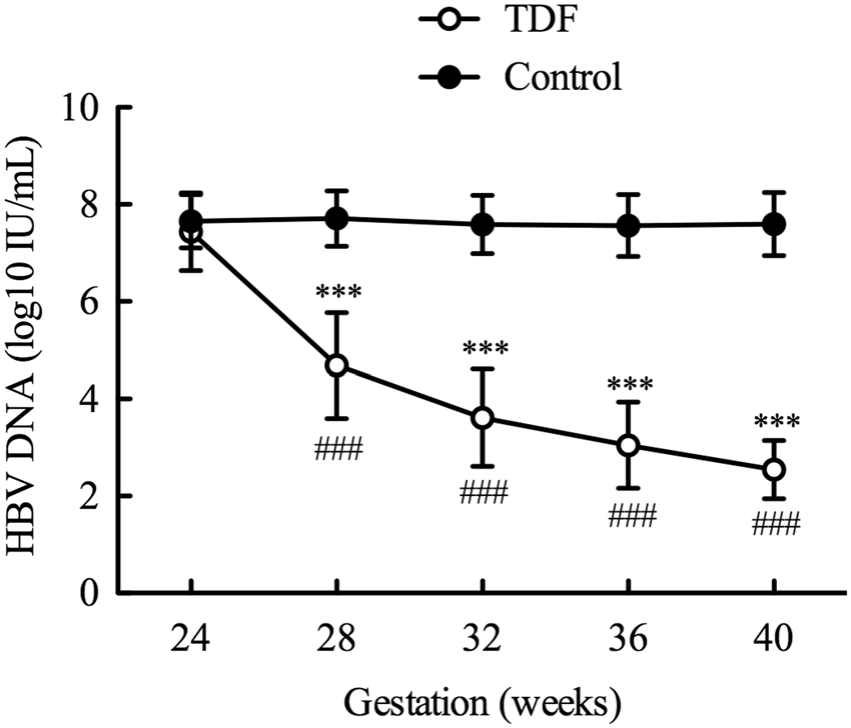
Changes in the serum HBV DNA levels over time. From 24 weeks of gestation, the pregnant women were treated with or without TDF (300 mg/day, oral). The serum HBV DNA levels were monitored every 4 weeks until delivery. ***p < 0.001 TDF treatment *vs*. control at the same time point; ###p < 0.001 compared to the DNA level at the previous time point in the TDF-treated women.

To evaluate the effect of TDF treatment on viral replication, the serum HBV DNA levels during the pregnancies were dynamically monitored every 4 weeks up to 28 weeks postpartum. As shown in Fig. 2 and the supplementary Table, prior to TDF treatment (24 weeks of gestation), the serum HBV DNA levels of the pregnant women were similar between the TDF-treated and control groups (7.44 ± 0.80 *vs*. 7.66 ± 0.55 log10 IU/mL, p=0.091). The HBV DNA levels in the control group exhibited no marked changes during the whole gestation period (Fig. 2, supplementary Table). Compared to that of the control group, TDF treatment significantly and gradually reduced the serum HBV DNA level (Fig. 2, supplementary Table).

Paired comparisons between the patients who received TDF treatment showed that the serum HBV DNA level decreased by 4.82 ± 0.94 log10 IU/mL from 24 weeks of gestation to delivery (p < 0.001, supplementary Table). By delivery, 90% of the patients with TDF treatment had a serum HBV DNA level of 2000 IU/mL or less; among them, 50% of the patients had HBV DNA levels less than 200 IU/mL.

The results also showed that the serum HBV DNA decreased rapidly after initiating TDF treatment. Four weeks after initiating TDF therapy, a sharp decrease in HBV DNA (2.75 ± 1.19 log10 IU/mL) was observed (7.44 ± 0.8 *vs*. 4.68 ± 1.09, p < 0.001) (Table 2 and supplementary Table). In addition, the serum DNA levels decreased continuously during TDF treatment (Fig. 2, Table 2). Although the velocity of the decline slowed with time, it was still maintained at approximately 0.5 log10 IU/mL per month after the 3^rd^ month (32 to 36 weeks) of treatment (Table 2), which kept the serum HBV DNA levels low during the remaining gestation period.

**Table 2:** The decrease in the HBV DNA levels after TDF treatment.

Compared time points	Mean	STD	p value*
24 *vs*. 28 weeks	2.75	1.19	<0.0001
28 *vs*. 32 weeks	1.08	0.92	<0.0001
32 *vs*. 36 weeks	0.57	0.59	<0.0001
36 *vs*. 40 weeks	0.49	0.58	<0.0001

^*^p < 0.05 indicates that the serum HBV DNA levels changed significantly between two time points.

We also evaluated the effect of TDF on mother-to-child transmission. The vertical transmission rate was determined in infants 28 weeks after birth by examining the serum HBV DNA levels (>20 IU/mL) or detection of HBsAg (positive). As indicated in Table 3, the TDF treatment group had no mother-to-child transmission events with HBV infection, whereas a vertical transmission rate of 13.5% (7/52 individuals) occurred in the control group (p = 0.004). Although one premature infant died at 32 weeks of gestation in the TDF treatment group, this preterm infant was later confirmed not to be infected with HBV.

**Table 3:** The mother-to-child transmission rates and the serum HBV DNA recovery of the mothers at 28 weeks postpartum.

	TDF treatment		Control		value	p
	Cases (n)	%	Cases (n)	%		
Mother-to-child Transmission					8.338	0.004
Occurred	0	0%	7	13.5%		
Did not occur	59	100%	45	86.5%		
Serum HBV DNA 28 weeks postpartum					21.916	0.000003
≤2000 IU/mL	20	33.9%	0	0%		
Recovery to the original level	39	66.1%	52	100%		

To examine HBV recovery after cessation of TDF treatment 4 weeks postpartum, the serum HBV DNA levels during the pregnancies were checked at 28 weeks postpartum. The results showed that 33.9% (20/59 individuals) of the TDF-treated individuals maintained serum HBV DNA levels less than 2000 IU/mL, whereas 66.1% of the patients (39/59 individuals) had recovered to their original HBV level (Table 3). Conversely, the HBV DNA levels in the control individuals did not change significantly before and after delivery and were always maintained at high levels compared to those of the TDF-treated patients, which was similar to their original levels and thus defined as 100% recovery (Table 3).

The emergence of drug-resistant variants was also evaluated by monitoring mutations of HBV genes in the TDF treatment and control groups using a direct gene sequencing technique. The results showed no gene mutations in HBV B2 or C2, which are the two major HBV strain genotypes in the Chinese population^29^.

The present study also investigated the outcomes of the mothers and children. As shown in Table 4, no significant differences were observed between the mothers in the TDF and control groups during pregnancy, delivery and cesarean section. One case of miscarriage (1.72%) occurred at 12 weeks of gestation in the TDF treatment group prior to TDF therapy. As described above, another case of preterm delivery occurred in the TDF-treated group at 32 weeks of gestation (1.69% of the preterm birth rate), and the preterm infant died 3 days after birth from hypoxemic brain disease without HBV infection. In the control group, 2 cases of preterm delivery (3.85%) occurred. However, no significant difference (p = 0.495) in preterm delivery was observed between the TDF and control groups (Table 4). In addition, 10.3% of the mothers from the TDF treatment group and 9.6% from the control group had cholestasis syndrome. No other obstetric complications were observed in either group. Furthermore, 3.45% of the patients from the TDF group (2 of 59 subjects) experienced nausea and vomiting, but they continued the TDF therapy with symptomatic treatment. No one in the control group had similar symptoms. Finally, approximately 10% of the patients had itchy skin in both the TDF and control groups (Table 4). No marked differences were observed between the groups in either digestive system or skin symptoms. No other adverse reactions appeared in the two groups of pregnant women.

**Table 4:** Maternal outcomes between the TDF treatment and control groups.

	TDF Group Mean ± SD or n (%)	Control Group Mean ± SD or n (%)	p
**Mothers**			
Pregnancy weeks	39.46 ± 1.43	39.33 ± 1.49	0.645
Cesarean section, n (%)	21 (36.2%)	17 (32.7%)	0.699
**Obstetric complications, n (%)**			
Pregnancy hypertension	0	0	
Placenta previa	0	0	
Intrahepatic cholestasis of pregnancy	6 (10.3%)	5 (9.6%)	0.899
Postpartum hemorrhage	0	0	
Limited fetal growth	0	0	
Preterm birth	1 (1.69%)	2 (3.85%)	0.495
Abortion	1 (1.72%)	0	0.342
**Adverse reactions, n (%)**			
Digestive tract reaction: vomiting	2 (3.45%)	0	0.177
Fatigue	0	0	
Headache	0	0	
Cough	0	0	
Fever	0	0	
Itchy skin	6 (10.3%)	5 (9.6%)	0.899
Jaundice	0	0	
**Laboratory examinations, n (%)**			
ALT, U/L >5 × ULN*	2 (3.45%)	0	0.177
Renal dysfunction	0	0	
Decrease of PLT	0	0	
Anemia	0	0	

^*^ULN: upper limit of normal.

None of the mothers in the present study breastfed their infants. A total of 58 healthy newborns were delivered in the TDF treatment group and 52 newborns were born in the control group, since 8 pregnancies were lost to follow-up during the study. Among all 110 surviving infants, 2 cases from the TDF treatment and 1 from the control group had mild asphyxia immediately after birth according to the Apgar score at 1 min (Table 5). No significant differences were found between the groups in the average body weight and length. Neither congenital malformations nor adverse symptoms were observed in any of the infants after birth. The serum ALT levels were similar in the infants in both groups. However, among the infants from the control group, 4 were HBsAg-positive (7.7%), 5 were HBeAg-positive (9.6%) and 7 cases (13.5%) had detectable HBV DNA (Table 5). None of the infants from the TDF-treated mothers were positive for HBsAg, HBeAg or HBV DNA, which was a marked difference compared to those of the control group (p < 0.05).

**Table 5:** Infant outcomes between the TDF treatment and control groups.

Infants			
Apgar score 1 min after birth, n (%)			0.613
8~10 normal	55 (96.5%)	51 (98.1%)	
4~7 mild asphyxia	2 (3.5%)	1 (1.9%)	
0~3 severe suffocation	0	0	
Weight (g)	3259.47 ± 369.03	3338.08 ± 363.04	0.265
Height (cm)	49.89 ± 0.82	49.92 ± 0.84	0.859
Congenital malformations, n (%)	0	0	
**Adverse reactions, n (%)**			
Fever	0	0	
Cough	0	0	
Vomiting	0	0	
Jaundice	0	0	
**Laboratory examinations, n (%)**			
ALT (U/L)	22.25 ± 11.92	24.27 ± 18.83	0.501
HBsAg-positive	0	4 (7.7%)	0.033
HBeAg-positive	0	5 (9.6%)	0.017
HBV DNA-positive	0 (0%)	7 (13.5%)	0.004

## Discussion

Benefiting from discovery of the HBV vaccine, the morbidity of HBV infection has decreased significantly. However, chronic HBV infection remains a large public health concern, particularly in the high endemic regions of Asia. Perinatal transmission is a major cause of this chronic infection. Indeed, HBV vaccine and immunoglobulin therapy have been shown to only prevent infection during the labor and postnatal periods but have no effect on intrauterine infection^30^. The HBV DNA level in pregnant women is also closely associated with the vertical transmission rate and immunoprophylaxis failure^19^. Furthermore, the repressed immune system during pregnancies^6,7^ also increases the potential risk of HBV proliferation during pregnancy. Thus, pregnant women with HBV infection, especially those who are HBeAg/HBsAg-positive with high viral loads, must control their viremia with antiviral treatment, such as TDF therapy, to decrease the risk of mother-to-child transmission of HBV and to maintain the health of the pregnancies. The EASL 2017 Clinical Practice Guidelines for the management of HBV infection recommended TDF treatment at 24–28 weeks of gestation during pregnancy^31^. However, limited data on the efficacy and safety of the application of TDF to control HBV infection during pregnancy are available, especially for the second trimester of gestation. The present study aims to investigate and evaluate the effect of TDF initiated at 24 weeks of gestation on the efficacy and safety, therapy duration, drug resistance and vertical transmission.

In the present work, we dynamically monitored the effect of TDF on the serum HBV DNA levels and found a total reduction of 4.82 ± 0.94 log10 IU/mL from 24 weeks of gestation to delivery (p < 0.001). Interestingly, a decline of 2.75±1.19 log10 IU/mL of HBV DNA was detected 4 weeks after TDF initiation. However, the pregnancies in the control group, which had similar viral loads to the TDF-treated pregnancies before initiating the therapy, had unchanged viral loads during gestation. In line with our finding, a gradual decrease in the serum HBV DNA level induced by TDF was observed in another study^28^, although the negative control (pregnancies without TDF treatment) was lacking in their study. All other clinical trials have only monitored the HBV viral burden before TDF treatment and after delivery. Our data are also intriguing because they show that after the third trimester (32 weeks of gestation), 90% of the patients with TDF treatment have serum HBV DNA levels of 2000 IU/mL or less until delivery and 50% have viral loads lower than 200 IU/mL. This finding suggests that TDF therapy initiated at 24 weeks of gestation can maintain the serum HBV DNA at lower levels during pregnancy until delivery, which may be the ideal condition to limit viral replication and ensure fetal growth.

Previous studies have demonstrated that initiation of TDF treatment at the third trimester (30–32 weeks) of gestation reduces perinatal transmission, but mother-to-child transmission of HBV infection still occurs^3,17,22–24^. As a prospective investigation, the current trial started TDF therapy at 24 weeks of gestation, and no vertical transmission was observed in the infants. Wang *et al*. showed a similar result, although a small sample of subjects (21 patients) was included in their study^28^. In another three trials, TDF was given as early as 5, 12 and 17 weeks of gestation, and no HBV-infected infants were observed^10,25,26^. However, these studies were retrospective studies that included small numbers of individuals with TDF treatment. These findings encouraged us to evaluate the efficacy and safety of TDF with more patients.

The present study and other trials from China^32,33^ have shown an immunoprophylaxis failure rate of up to 15% in infants born to HBsAg-positive mothers in the control group. However. clinical trials from other countries, such as Thailand, showed a lower rate of immunoprophylaxis failure (<5%)^34^. The following factors may account for this discrepancy: 1) the timeframe of immunoprophylaxis administration; indeed, the infants received immunoprophylaxis at approximately 1 hour after birth in the study from Thailand compared to vaccination of infants within 12 h after birth in China; 2) the vaccine dose; the routine vaccine series (3 doses at 0, 1 and 6 months after birth) are applied in China, whereas infants receive 5 doses of the vaccine (0, 1, 2, 4 and 6 months) in Thailand; and 3) HBV DNA levels of the mothers; trials from various countries have shown that immunoprophylaxis failure in infants is closely associated with the mother’s viral load at delivery^35–37^. Jourdain *et al*. also declared that the mothers of HBV-infected infants from Thailand had high viral loads at delivery^34^.

In addition to the decline in the serum HBV DNA levels induced by TDF treatment, TDF therapy was also reported to result in seroclearance of HBeAg and HBsAg or HBeAg seroconversion^17,22,26^. Marcellin *et al*. even showed that HBeAg could not be detected in 45% of patients after TDF treatment^26^, which might be associated with the HBeAg levels prior to initiation of TDF therapy. However, the present study did not observe any seroclearance/seroconversion for either HBeAg or for HBsAg in either the TDF-treated or control mothers. The potential reasons are that all pregnancies in our study 1) have a high titration of HBeAg and HBsAg with a high viremia and 2) are tolerant to HBV infection since they have high viral loads (>2×10^6^ IU/mL) and a relatively normal ALT level.

Concerning the safety of TDF treatment, we did not detect any congenital malformations during gestation and at 6 months after birth. A previous study revealed that a high dose of TDF but not the dose used for humans affected fetal growth in primates^38^. Indeed, TDF treatment of pregnancies with chronic HBV infection has been associated with adverse events, including a low birth weight, premature delivery and congenital abnormalities, regardless of whether TDF is given at the second or third trimester of gestation^3,10,17,23,24^, although these side effects are much lower than those induced by other NAs. The current study also observed two premature deliveries at 33 and 34 weeks of gestation from the control group, of which both infants survived. However, a preterm delivery occurred in the TDF-treated group, and the infant died from hypoxic encephalopathy; we found no evidence of a direct association with TDF treatment, although we could not exclude this possibility. Therefore, a large amount of complementary data are still needed to further evaluate the safety of TDF on pregnancies and infants. Because the children were followed up to 28 weeks after birth in the present study, the long-term or delayed potential effects of TDF on the infants (e.g., growth and intelligence) also need to be addressed in the future. TDF-treated mothers have breastfed their children without reported complications^26^. However, according to the guidelines from the Chinese Foundation for Hepatitis Prevention and Control, breastfeeding should not be recommended since the potential risk for the infants is still uncertain; further studies are needed to clarify the safety.

Drug resistance or tolerance is a big problem during chronic hepatitis B infection; for instance, a Lamivudine (LAM)-resistant viral variant has been reported after short-term therapy^39^. Our present study did not detect resistant variants of HBV even when we started treatment at 24 weeks of gestation and continued it to 4 weeks postpartum. Consistent with our observation, other studies also did not show the emergence of resistant variants of HBV during TDF therapy at the third trimester of gestation of pregnancy^3,17,22,24^, which might be due to the high resistance barrier of TDF^5^.

In the present trial, the women stopped TDF treatment at 4 weeks postpartum. Notably, 33.8% of the subjects maintained lower serum HBV DNA levels under 2000 IU/mL at 28 weeks postpartum, whereas the HBV DNA level recovered to the original level in the remaining individuals. In addition, only 3.4% of the TDF-treated patients had liver function more than 5 times the upper limit of normal without other clinical manifestations. No patients had kidney dysfunction in TDF treatment group. In line with our observations, Nguyen *et al*. reported that regardless of whether TDF was immediately withdrawn after delivery or continuously used, this medicine did not affect the risk of HBV DNA recovery in pregnancies^40^. However, another study recommended close monitoring up to 6 months after delivery for women who were HBeAg positive or had stopped TDF treatment^41^. Nevertheless, our study only followed up the patients to 28 weeks postpartum, and thus further intensive monitoring and follow-up are needed.

In summary, initiation of TDF treatment at the second trimester (we started at 24 weeks) of gestation in HBsAg/HBeAg-positive pregnant women with high viral loads (≥2×10^6^ IU/mL) dynamically and efficiently reduced the serum HBV DNA levels. This reduction was apparent at the first month of TDF therapy, which facilitated maintenance of HBV DNA at the lower level during the following gestation periods of the pregnancies. Treatment apparently reduced the risk of vertical transmission and ensured an optimal uterine microenvironment for the fetus. Our results demonstrated that application of TDF at 24 weeks of gestation in HBV-infected pregnancies was safe and that no obvious complications for the mothers, severe outcomes or adverse effects were observed. These results indicate the efficacy and safety of TDF therapy at the middle stage of gestation in HBV-infected pregnancies, particularly for those who have high viral loads and are HBsAg/HBeAg-positive, to prevent mother-to-child transmission.

## Methods

### Study design and research subjects

The multicenter cohort study was performed from January 2013 to December 2016 in different hospitals located in northwest China. The trial was approved by the Ethics Committee of the First Affiliated Hospital of Xi’an Jiaotong University (Xi’an, China), and the date of registration was March 25, 2016 (registration number: NCT02719808). The study was performed in accordance with the guidelines and regulations for the prevention of vertical transmission of hepatitis B in pregnancy (Chinese Medical Association-Society of Obstetrics and Gynecology, 2013). The details of the full protocol are available in the online complementary data section. The flow chart of study subject selection is shown in Fig. 1. The eligibility criteria were 20–35-year-old pregnant women who were HBeAg/HBsAg double-positive with a serum HBV DNA titer ≥2×10^6^ (6.3 log10) IU/mL. The exclusion criteria for the subjects were ① co-infection with HIV, HCV or HDV, ② a HBV treatment history within 6 months, ③ an abortion history or clinical manifestation of an inevitable abortion, ④ congenital deformity of the fetus, ⑤ evidence of hepatocellular carcinoma, renal or hepatic dysfunction, a creatinine clearance rate <100 mL/min, ALT >5 times the upper limit of normal, or bilirubin >2 mg/dl, ⑥ hemoglobin <8 g/100 mL, neutrophils <1000/mm^3^, or albumin <2.5 g/100 mL, ⑥ special medicine treatment required during the pregnancy, and ⑦ the biological father of the infant has chronic HBV infection. A total of 120 women were finally enrolled in the study according to the criteria. All participants were informed about the study, volunteered and signed the written consent form.

The parallel study was designed with TDF-treated and control groups. The sample size was determined using an online sample size calculator for two parallel-sample proportions. A random number table was used to group the pregnancies into each group (60 individuals per group) based on their enrollment time. Simple randomization was performed, and sealed envelopes were used for concealment of the random allocation. TDF treatment (300 mg/day, oral, GSK, China) was initiated at 24 weeks of gestation and continued to 4 weeks postpartum. The control individuals did not receive anti-viral treatment. All subjects were followed up every 4 weeks to 28 weeks postpartum, and the laboratory parameters were monitored at every visit. All infants were given routine immunoprophylaxis, such as hepatitis B immune globulin (HBIG), immediately after birth and the HBV vaccine within 12 hours after birth. The second and third doses of the vaccine were given to the infants at ages 1 and 6 months, respectively. Due to insufficient long-term safety data for infants exposed to TDF through breastmilk^24^, none of the infants were breastfed according to the guidelines from the Chinese Foundation for Hepatitis Prevention and Control. The infants were also monitored from their birth to 7 months of age. During the double-blind study, the participants did not know which type of intervention they accepted until the end of the intervention. The participants, care providers and persons who examined the viral DNA loads and evaluated the outcomes of the patients did not know whether the patients had accepted the intervention.

### Laboratory evaluations

In accordance with the updated Chinese guideline on “management algorithm for interrupting mother-to-child transmission of hepatitis B”^42^, serological testing was performed in the infants 28 weeks after birth (one month after the final dose of the HBV vaccine). The occurrence of mother-to-child transmission was the primary outcome and was determined when the serum HBV DNA of the infant was higher than 20 IU/mL or the infant was HBsAg-positive 28 weeks after birth. Congenital malformations were defined as anatomical abnormalities of the fetus before delivery or of infants 28 weeks after birth and were verified by clinical examinations, radiographic examinations or other experimental methods during follow-up. The rate of congenital malformations reflects the proportion of infants with defects out of all safe-born infants.

To evaluate the effect of TDF, the serum HBV DNA level was monitored in all pregnancies with/without TDF treatment. The assay was performed before initiating TDF therapy and then continued every 4 weeks until immediately before delivery. After delivery, this procedure continued to 28 weeks postpartum to evaluate recovery of HBV DNA after cessation of TDF treatment, which was defined as the secondary outcome.

Total DNA was extracted from the patient’s blood using the DNAout kit (Tianenze, Beijing, China). The RT region (NT 54–1278) was amplified (Chinese patent ZL 200910092331.1) as described previously^43^. Briefly, the sense and antisense primers for the first-round PCR were 5′-agtcaggaagacagcctactcc-3′ (NT 3146–3167) and 5′-aggtgaagcgaagtgcacac-3′ (NT 1577–1596), respectively. The primers for the second-round PCR were 5′-ttcctgctggt-ggctccagttc-3′ (NT 54–75) and 5′-ttccgcagtat-ggatcggcag-3′ (NT 1258–1278). The PCR products from all patients were sequenced to check whether gene mutations occurred after TDF treatment during the pregnancy period.

In the present study, ALT above 5 times the upper limit of normal was identified as a significant adverse event clinically regardless of whether the patients had symptoms. These patients needed to be further monitored and followed up or treated.

### Statistical analysis

The data were presented as the mean±SE, and SPSS 18.0 (SPSS Inc., Chicago, IL, USA) was used to conduct the analysis. Baseline characteristics and safety outcomes were compared between the TDF treatment and control groups using t-tests for continuous variables and the Chi-square test or Fisher’s exact test for categorical variables. The mother-to-child transmission rate and the recovery level of the HBV DNA 28 weeks after delivery were compared between the two groups using Fisher’s exact test. A mixed model repeated measures analysis of variance was used to analyze whether the serum HBV DNA level changed differently with or without TDF treatment. Planned contrast was used to test the “change in HBV DNA for each group between baseline and delivery time” and “differences from week to week’ within the TDF treatment group. A p value less than 0.05 was considered statistically significant.

## Electronic supplementary material

Supplementary table

Supplementary information

## Data Availability

The protocol used for this study is included in this published article (Supplementary files). All data generated or analyzed during the study are available from the corresponding author on the reasonable request.
