# Supplementary material for: Efficacy of tenofovir in preventing perinatal transmission of HBV infection in pregnant women with high viral loads
Source: Sci Rep. 2018 Oct 19;8:15514. doi: 10.1038/s41598-018-33833-w (PMC6195597; doi:10.1038/s41598-018-33833-w)
Supplement: Supplementary file 1 — Supplementary table [file 41598_2018_33833_MOESM1_ESM.doc]

Efficacy of tenofovir in preventing perinatal transmission of HBV infection in pregnant women with high viral loads

Yayun LIN^1^, Yan LIU^2^, Guifeng DING^3^, Lhousseine TOUQUI^4^, Weimin WANG^1^, Na XU^1^, Keying LIU^1^, Lingyan ZHANG^5^, Dunjin CHEN^6^*, Yongzheng WU^7^*, Guiqin BAI^1^*

1 Department of Gynecology and Obstetrics, The First Affiliated Hospital of Xi’an Jiaotong University, Xi’an

2 Research Center for Clinical and Translational Medicine/Institute of Infectious Diseases, Beijing 302 Hospital, Beijing

3 Department of Obstetrics, Maternal and Child Health Care Hospital of Xinjiang Uygur Autonomous Region, Urumqi

4 Equipe Mixte Institut Pasteur/Paris V, Department of Infection & Epidemiology, Institut Pasteur, Paris, France

5 Department of Gynecology and Obstetrics, ShaanXi Provincial People Hospital, Xi’an

6 Third Affiliated Hospital of Guangzhou Medical University, Guangzhou

7 Unit of Cellular Biology of Microbial Infection, CNRS UMR3691, Institut Pasteur, Paris, France

**SUPPLEMENTARY TABLE:**

Table S. HBV DNA level (log10 IU/mL) by treatment group and time point§

| Week | Tenofovir (N=59) | Control (N=52) |
| --- | --- | --- |
| 24 | 7.44±0.80 | 7.66±0.55 |
| 28 | 4.68±1.09 | 7.71±0.57 |
| 32 | 3.61±1.00 | 7.59±0.60 |
| 36 | 3.05±0.89 | 7.57±0.64 |
| 40 | 2.54±0.60 | 7.60±0.65 |

^§^HBV DNA level was expressed as log_10_ IU/mL.
