# Supplementary material for: Efficacy of tenofovir in preventing perinatal transmission of HBV infection in pregnant women with high viral loads
Source: Sci Rep. 2018 Oct 19;8:15514. doi: 10.1038/s41598-018-33833-w (PMC6195597; doi:10.1038/s41598-018-33833-w)
Supplement: Supplementary file 2 — Supplementary information [file 41598_2018_33833_MOESM2_ESM.docx]

**CLINICAL TRIAL**

Title: the prospective study in investigating the efficacy of Tenofovir in preventing perinatal transmission of HBV infection in pregnant women with high viral loads

Date: Novembre 20, 2012

Intervention: tenofovir disoproxil fumarate (TDF)

Form and route tablet, oral

of administration:

Manufacturer: GSK®, China

Principle Guiqin BAI, professor

Investigator: Department of Genecology and Obstetrics, The First Affiliated Hospital of Xi’an Jiaotong University, YanTa West Road No.277, Xi’an 710061, Shaanxi, China.

**Abstract*:**

After more than 20 years of studies on the mother-to-child transmission and the therapy strategies against hepatitis B virus (HBV) infection, we have known that the HBsAg/HBeAg and HBV DNA level are the predominant factors related to the HBV intrauterine infection. The present project will prospectively investigate the effect of tenofovir disoproxil fumarate (TDF), a nucleotide analogue against viral infection, in blocking the HBV vertical transmission and the potential mechanism involved in this process among the pregnant women who are HBsAg/HBeAg-positive with high viral loads. The trial will be randomly designed. We will check the efficacy and rationality of the TDF in blocking the mother-to-child transmission of HBV infection (primary outcome). We will dynamically monitor the HBsAg/HBeAg, HBV DNA levels (secondary outcome) and liver function of the women during the pregnancy and the hepatitis B parameters, HBV DNA level (secondary outcome) and liver function of infants immediately after the birth and at age of 7 months. The ratio of T lymphocytes and cytokines level in the serum related to the HBV infection will also be checked (before TDF intervention, before and after delivery, etc) to reveal the effect of TDF on the immune function of the pregnancies, which will help to determine whether the changes of immune system by TDF contribute to the block of vertical transmission of HBV. The known and/or potential mutation site(s) of genes related to the drug resistance or tolerance will be examine by PCR in the pregnancies after TDF treatment, which will facilitate the subsequent therapy against chronic HBV infection. The infants from TDF treated mothers will be followed up till 2 years old and monitor the growth curve and IQ value. In summary, the present study will allow us to evaluate the efficacy and safety of TDF in the pregnancies and to provide the evidence of TDF against HBV during the pregnancy.

*** Since the original protocol was wrote in Chinese and there are too much contents, I try my best to translate the key points of the protocol into English.**

**INTRODUCTION:**

Hepatitis B virus (HBV) infection is a main problem for the public health, particularly, in the endemic regions, such as in China. It is estimated that 9.9% of the population in China are HBV carrier and that approximately 120 millions of patients are chronic HBV infection. The mother-to-child transmission is considered as the main cause of chronic HBV infection, which can occur in the utero, during child birth, and postpartum.

The application of hepatitis B vaccine and hepatitis B immunoglobulin (HBIG) effectively blocks the infant infection during the delivery process and postpartum after the birth from the HBsAg-positive mother. But it has no effect on the intrauterine infection that contributes to 5-8% of HBV infection. However, with the improvements of the research methods and detection sensitivity, the intrauterine infection has been shown to contribute to 40-60% of the HBV infection. It believes that the chronic HBV infection still occurs in 5-15% of infants born to the HBeAg-positive mother even regular immunoprophylaxis is given. Therefore, the intrauterine HBV infection is still one of the important reasons for the immunoprophylaxis failure. Thus, it is critical to block the intrauterine infection in order to control the hepatitis B epidemic and to prevent the HBV-related diseases in China and worldwide.

Ten years ago, multiple injections of HBIG was used in the pregnant women at the late gestation to block the mother-to-child transmission of the HBV infection. But the effect is not clear and the guidelines are no longer recommended. Nowadays, no available guideline is used around the world to block HBV intrauterine infection during the pregnancy. The studies have demonstrated that HBV DNA and HBxAg are high risk factors for HBV intrauterine infection. So, control of the HBV DNA levels and the HBXAG expression will be expected to attenuate HBV intrauterine infection.

The nucleoside analogues (NAs) have been widely used against viral infection such as HIV and HBV. The NAs play their roles by mimicking the structure of nucleotides, but do not function as the nucleotides. Therefore, the NAs incorporate into HBV DNA during the process of DNA synthesis and synthesize the non-function nucleic acid chain, leading to the termination of the replication of hepatitis B virus. Among the NAs, lamivudine and Biff have higher incidence of drug resistance; the antiviral effect of adefovir ester is weaker; entecavir and tenofovir have stronger antiviral effect with the lower resistance rate. Based on the safety for the pregnancy, the FDA of USA categorizes tenofovir and Biff as "B" class reagents, since their safety have been approved in the animal model but limited data in the human application. The animal study also shows that lamivudine, adefovir and entecavir result in teratogenic embryo. Thus, they are categorized as "C" class reagent by the FDA, although no data from humans for these medicines. In addition, the global multicentre clinical trial and clinical study in China have shown that the application of lamivudine at late stage of gestation can inhibit the HBV replication, reduce viral load in the pregnant women, recover ALT and HBeAg to the normal level, thus decrease the intrauterine infection of HBV. However, lamivudine is confirmed to have high risk in inducing viral mutation and belongs to the "C-class" medicine for the pregnancy, thus generally it is not recommended for the pregnant women. The study on the use of adefovir ester in the pregnant women is inadequate.

Tenofovir (tenofovir disoproxil fumarate, TDF), a new type of nucleotide reverse transcriptase inhibitors, is effectively against a variety of viruses therefore used for the treatment of viral infectious diseases. The TDF component tenofovir diphosphate inhibits the viral polymerase by competitively combining with the substrate of natural deoxyribose. As mention above, it can also terminate the DNA chain synthesis by inserting into DNA chain. The in vitro studies have shown that TDF is effective against a wide range of viruses including the strains that are resistant to nucleoside reverse transcriptase inhibitors. Van Bommel reported that 10 patients, who were chronic HBV infection and tolerated to lamivudine (LAM) and subsequent adefovir therapy, have been treated only with TDF for more than 12 months and no drug resistance was found in all those patients, suggesting that unique TDF treatment has significant antiviral effect, particularly, for the patients tolerating to the other NAs medicines.

As a “B” class medicine, the TDF has been approved for its safety and efficacy in primates. Until today, there is only one report about its effect on the intrauterine HBV infection. In this study with small sample size (8 subjects), the pregnant women accepted 300mg/day TDF orally. Among those patients, one case was pregnant during TDF treatment; 3 individuals took TDF to control active hepatitis; 4 patients were given TDF to prevent intrauterine HBV infection. All those patients continued to accept TDF therapy after the delivery. Their results showed that patents’ HBV DNA levels decreased 2-4 log10 copies/mL in the first 4 weeks and were less than 10^4^ copies/mL after the delivery. They suggested that the application of TDF at late stage of the gestation was safe and did not induce the drug resistance. In addition, given that the TDF efficiently inhibits the viral DNA replication with limited kidney cytotoxicity, more and more studies focus on the TDF antiviral treatment in the pregnancies at late stage of gestation. Indeed, compared to other NAs, TDF has not been reported for the drug resistance even after 3 years treatment. However, more studies with large size of the subjects and longer duration of follow-up are necessary to further confirm the safety and efficacy of TDF in pregnant women.

**HYPOTHESIS**

Based on the evidences described above, the TDF effectively inhibits the HBV replication with less renal toxicity. Particularly, it is also effective in the patients tolerating to other NAs treatment and no drug resistance is reported. Intriguingly, the TDF has been tested in the pregnancies at the late stage of gestation against HBV infection. However, the prospective studies with large sample size and control populations are lacked to confirm definitely the safety and efficacy of TDF on the pregnant women and infants.

Therefore, the present trial is designed to prospectively investigate the effect of TDF in the pregnancies. The pregnant women will be randomly grouped into TDF treatment and control subjects. To test its maximal effect, the patients who are HBsAg/HBeAg positive with high viral burdens will be selected, since, as mentioned above, the HBxAg and HBV DNA levels are closely associated with intrauterine HBV infection. On the other hand, the application of TDF at late stage of the gestation might be too late to block intrauterine HBV infection, particularly, in the pregnancies who are HBsAg/HBeAg-positive with high viral burdens. Thus, we propose to give TDF treatment at the mid stage of gestation (24 weeks) to inhibit the intrauterine infection by HBV.

**STUDY OBJECTIVES**

The present prospective study aims at investigating the effect of TDF against the mother-to-child transmission of HBV infection. Through this trial, we will:

(1) confirm the efficacy of TDF in blocking HBV intrauterine infection during pregnancy;

(2) monitor the effect of TDF on the dynamic changes of HBV DNA levels in the pregnancy;

(3) test the HBsAg and HBeAg expression during and after the TDF treatment;

(4) screen the changes of HBV resistance genes related to the NAs treatment in the pregnant women with chronic HBV infection;

(5) clarify the adverse effects induced by TDF treatment during the pregnancy for both mother and infants.

This clinical project will allow us to better understand the safety and efficacy of TDF against HBV infection during the pregnancy. It will also help in clarifying the potential adverse effects of this medicine. The study will also provide the evidence to further optimize and to facilitate the TDF application in blocking the vertical transmission of HBV infection.

**INTERVENTION**

The TDF is manufactured as tablet and will be taken orally. The dose will be 300mg/day.

The therapy will be performed in three hospitals of China (The First Affiliated Hospital of Medical College of Xi’an Jiaotong University, Shaanxi Provincial People Hospital and Maternal and Child Health Care Hospital of Xinjiang Uygur Autonomous Region). The study will last for 4 years from January 2013 to December 2016.

The TDF therapy will be started at 24 weeks of gestation. To evaluate the effect of TDF, the blood sample will be collected in all the pregnancies with/without TDF treatment before initiating the TDF therapy, then every 4 weeks till just before delivery. After delivery, this procedure continues till 28 weeks postpartum. After collecting the blood, the parameters related to live function and HBV infection will be checked within 24h. The aliquot of blood will be used to extract DNA using DNAout kit to check the expression of HBxAg and the mutation of drug resistant genes. The left will be used to harvest serum. In brief, the blood will be kept in ice for 30min before the centrifugation. The serum will be aliquoted and kept at 80°C for the further analysis. All sample tubes will be labelled with the case number of patient.

**STUDY DESIGN**

A random study will be designed to investigate the effect of TDF in blocking vertical transmission of the HBV infection.

Before starting the trial, we estimate the sample size using online sample size calculator for two parallel-sample proportions (http://clincalc.com/stats/samplesize.aspx). According to the literatures, we learn that after routine immunoprophylaxis the mother-to-child transmission of HBV infection is around 10-30% (we proposed as 30% for the calculation). We expect the TDF treatment will reduce the infection to 5-10% (we perform calculation as 5%). The superiority margin is supposed to be 0.05. So, we have an estimated samples size with equal allocation as 35 for each group. The present trial will collect 60-80 individuals for each group.

We will randomly allocate patient number into TDF and control group based on the random number table. The patients for the study will be selected as shown in the bellowing flow chart. When the pregnant women visit the hospital for the first time, generally, the routine care will be given and the laboratory parameters of blood sample including blood cell counts, HBV parameters, liver function, kidney function, etc. will be checked. The ultrasound of foetus will also be performed. Based on the laboratory data and history, the non-eligible pregnancies will be excluded. The eligible pregnant women will be further selected according to the criteria. Then, the final enrolled pregnancy will be numbered as the visiting order. This patient number will be corresponded to the allocated number from random number table.

During the study, the patient will be excluded if special situation occurs including preterm delivery, lost follow up or any other severe side effects.


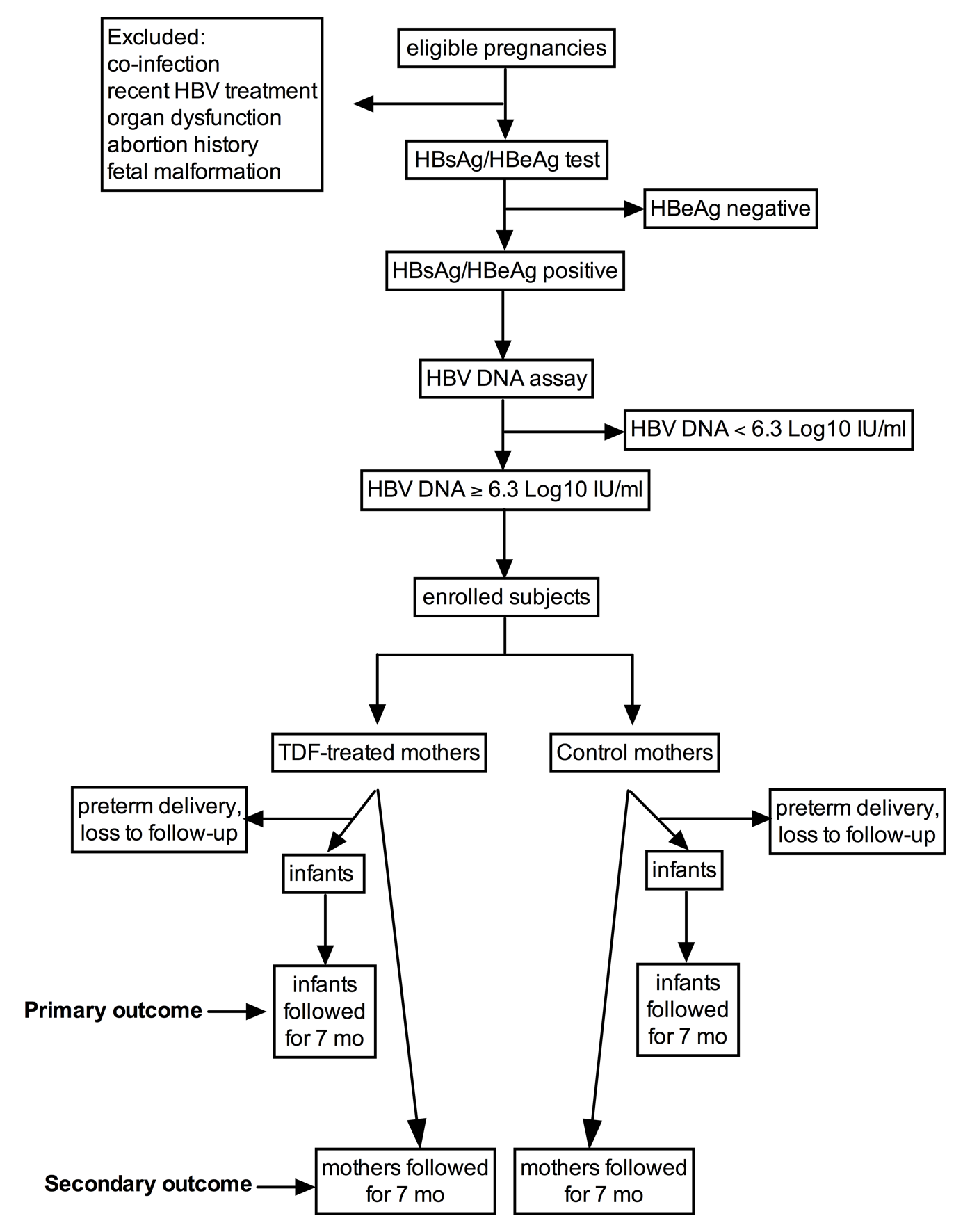


- Subject:

According to our experiences, it is estimated that around 80-150 pregnancies with HBV infection will visit our hospitals. Three hundred to 500 HBV-infected pregnant women totally will be predicted in 3-4 years.

- Eligibility criteria

20-35 years old pregnant women with the HBV infection.

- Inclusion criteria

The pregnancies who are HBeAg/HBsAg double positive as well as serum HBV DNA≥2×106 (6.3 log10) IU/mL.

- Exclusion criteria

① co-infection with HIV, HCV or HDV; ② HBV treatment history within 6 months; ➂ abortion history or clinical manifestation of inevitable abortion; ④ congenital deformity of foetus; ⑤ hepatocellular carcinoma evidence, renal or hepatic dysfunction, creatinine clearance rate <100mL/min, alanine aminotransferase (ALT) >5 times of upper limit of the normal level, bilirubin >2mg/dl; ⑥haemoglobin <8g/100mL, neutrophil <1000/mm^3^, albumin <2.5g/100mL; ⑥ special medicine treatment during the pregnancy; ⑦ biological father of infant has chronic HBV infection.

- Randomization

Based on our prediction, less than 50 pregnancies per year are eligible for the study. To ensure the similar number for treatment and control group, we estimate that minimal 30 subjects can participate in the study (total around 120 subjects). When getting 120 subjects, the enrolment will be stopped.

In random number table, we randomly start and separate 3 digital number as group following the order in number table. The separated number are written on the paper following the order of the random number table. The repeated number and number big than 120 will be excluded. The first 60 number based on the order will be allocated as TDF treatment. The left will be controls.

- Conceal the intervention

The selected number and corresponding intervention will be hided in the individual envelope and sealed. On the surface of the envelop, the selected number is written. Then the envelops are managed in order (from 001 to 120) and given to the doctor when having the enrolled subject (visiting order).

- Ethics

We have got the approval from the hospital for the study (approval N° 2012-136). The consent will be prepared and printed. The patients will be informed and sign the written consent.

- Potential benefit to the subjects

The TDF therapy in the HBV-infected pregnant women with high viral loads will help in reducing the risk of mother-to-child transmission. It will also protect mothers during the gestation periods due to the special immune situation. In addition, it will help to understand the safety and efficacy of TDF in blocking the vertical transmission, its effect on mothers and infants and potential adverse effects, which will facilitate the TDF treatment in the pregnancies in clinic.

- Potential risk

The most potential risk for the trial is the side effects of TDF, such as preterm delivery and severe adverse effects including dysfunction of liver and kidney as well as the HBV resistance to TDF treatment. The trial has no apparent physical, psychological and economic effect. The pregnancies will not have any stress or discomfort when answering the question or focusing on the research. The participants will face the same stress as other pregnancies when collecting blood routinely.

- Methods to protect the privacy, data and right of subjects

Before starting the trial, the participants will be informed, agree and sign the printed consent. All the personal information will be confidential. A person will specifically manage the collected data that will be saved in the computer with password. Only this person can access the computer.

- Intervention

The TDF therapy will be given to the pregnant women who are HBsAg/HBeAg-positive with high viral burdens. The details of the medicine will be concealed. The pregnancies will take the medicine orally, 300mg/day, starting the date of enrolment (24 weeks of gestation) until 4 weeks postpartum. This dose is recommended by the manufacture and also used by most of the studies. The method used for assigning subjects to the treatment group is described above in the section of randomization.

The medicine will be distributed to the participants every time when visiting the doctor. The medicine will be prepared and packaged in the pharmacy centre of the hospitals.

**STUDY PROCEDURES**

- Screening for eligibility

The participants will be screened by the consulting doctor when they visit the hospital for the first time based on the criteria. The potential eligible pregnancies will be monitored every 4 weeks till 24 weeks of gestation. According to the parameters of pregnancies at 24 weeks of gestation, the pregnant women will be enrolled or not for the study.

- Visit

The enrolled pregnant women will visit the doctor every 4 weeks till 28 weeks postpartum.

- Data collection

Routinely, when patients visit doctor for the first time, all personal information will be recorded as electronic file including birth date, home address, contact information, family condition (family members, estimated annual income, work condition, etc), health history, etc. The medical records from other hospital(s) will also be collected.

The consulting doctor will provide the prescription for the lab examination or other examination such as ultrasound and infant monitor. All the obtained results from the examination will send back to the doctor. The person who is charge of the study will record all data in the specialized computer.

- Outcomes measurement

The primary outcome is the mother-to-child transmission of HBV infection, which will be determined when the infant’s serum HBV DNA is higher than 20 IU/ml or HBsAg is positive 28 weeks after the birth.

The secondary outcome is the recovery of HBV DNA levels at 28 weeks postpartum in the pregnancies with or without TDF treatment after the cessation of TDF treatment.

- Safety and adverse event monitoring

The doctor will carefully ask the condition and situation of the pregnancies during the consultation. At the same time, the safety is also monitored by the blood assay.

Based on the severity of patients’ situation (vomit, rash on the skin, liver and kidney function, etc), the doctor will give the corresponding treatment and decide whether withdrawing the study for the patient.

The emergence contact information will be provided to the pregnancies in case needing help and suggestion from doctor as well as reporting the adverse events. All the adverse effect will be recorded till the complement of the follow-up.

**STATISITICAL ANALYSIS**

- All data will be collected together to the person who is in charge of data collection. The data will be save as Excel file format.
- When the study is finished, we will discuss with statistician from the host university in managing and analyze the data.
- The data will be presented as mean±SE and SPSS18.0 (SPSS inc., Chicago, USA) will be used for the analysis. Baseline characteristics and safety outcomes will be compared between the TDF treatment and the controls using t-tests for continuous variables and chi-square tests or fisher exact tests for categorical variables. The Mother-to-child transmission rate and the recovery levels of the HBV DNA 28 weeks after the delivery between the two groups will be compared by Fisher exact tests. A mixed model, repeated measures analysis for variance will be used to analyze if the serum HBV DNA levels change differently with or without TDF treatment. The planned contrasts will be tested for the "change in HBV DNA for each group between baseline and delivery time" and "differences from weeks to the weeks’ within TDF treatment group. The p value less than 0.05 will be considered as statistically significant.
- All data will be confidential during and after the study. The data will be transferred and stored in the server of the hospital. Special authorization is needed to access these data after the closure of the study.

**STUDY ADMINISTRATION**

This study is coordinated by the Department of Obstetrics and Genecology, The First Affiliated Hospital (Xi’an Jiaotong Univerity Medical College). The leading PI is Pr Guiqin BAI. The Department of Obstetrics from The Maternal and Child Health Care Hospital of Xinjiang Uygur Autonomous Region and the Department of Genecology and Obstetrics from ShaanXi Provincial People Hospital will also participate in the trial.

**PUBLICATION**

After the study, we plan to write one or two manuscripts for the publication. The results will also be used for the financial funding application.

**REFERENCES**

- [Tong MJ, Thursby M, Rakela J, et al. Studies on the maternal-infant transmission of the viruses which cause acute hepatitis. Gastroenterology 1981; 80:999.](http://www.uptodate.com/contents/hepatitis-viruses-and-the-newborn-clinical-manifestations-and-treatment/abstract/1)
- [叶峰](http://acad.cnki.net/kns55/popup/knetsearchNew.aspx?sdb=CJFQ&sfield=%e4%bd%9c%e8%80%85&skey=%e5%8f%b6%e5%b3%b0&scode=09095173%3b09075374%3b09074653%3b08445637%3b09341336%3b11124277%3b27699111%3b09124521%3b09093877%3b10184883%3b10185648%3b); [蔺淑梅](http://acad.cnki.net/kns55/popup/knetsearchNew.aspx?sdb=CJFQ&sfield=%e4%bd%9c%e8%80%85&skey=%e8%94%ba%e6%b7%91%e6%a2%85&scode=09095173%3b09075374%3b09074653%3b08445637%3b09341336%3b11124277%3b27699111%3b09124521%3b09093877%3b10184883%3b10185648%3b); [金燕](http://acad.cnki.net/kns55/popup/knetsearchNew.aspx?sdb=CJFQ&sfield=%e4%bd%9c%e8%80%85&skey=%e9%87%91%e7%87%95&scode=09095173%3b09075374%3b09074653%3b08445637%3b09341336%3b11124277%3b27699111%3b09124521%3b09093877%3b10184883%3b10185648%3b); [师娟子](http://acad.cnki.net/kns55/popup/knetsearchNew.aspx?sdb=CJFQ&sfield=%e4%bd%9c%e8%80%85&skey=%e5%b8%88%e5%a8%9f%e5%ad%90&scode=09095173%3b09075374%3b09074653%3b08445637%3b09341336%3b11124277%3b27699111%3b09124521%3b09093877%3b10184883%3b10185648%3b); [邱洪涛](http://acad.cnki.net/kns55/popup/knetsearchNew.aspx?sdb=CJFQ&sfield=%e4%bd%9c%e8%80%85&skey=%e9%82%b1%e6%b4%aa%e6%b6%9b&scode=09095173%3b09075374%3b09074653%3b08445637%3b09341336%3b11124277%3b27699111%3b09124521%3b09093877%3b10184883%3b10185648%3b); [张曦](http://acad.cnki.net/kns55/popup/knetsearchNew.aspx?sdb=CJFQ&sfield=%e4%bd%9c%e8%80%85&skey=%e5%bc%a0%e6%9b%a6&scode=09095173%3b09075374%3b09074653%3b08445637%3b09341336%3b11124277%3b27699111%3b09124521%3b09093877%3b10184883%3b10185648%3b); [孔颖](http://acad.cnki.net/kns55/popup/knetsearchNew.aspx?sdb=CJFQ&sfield=%e4%bd%9c%e8%80%85&skey=%e5%ad%94%e9%a2%96&scode=09095173%3b09075374%3b09074653%3b08445637%3b09341336%3b11124277%3b27699111%3b09124521%3b09093877%3b10184883%3b10185648%3b); 胚胎中HBV mRNA的表达与父婴传播关系的研究 中华男科学杂志，2013,19（5）：429-433
- Lin HH, Lee T, Chen D, et al. Transplacental leakege of HBsAg-positive maternal blood as the most likely route in causing intrauterine infection with hepatitis B virus. J Pediatr, 1987, 111:877-881.
- 闫永平，徐德忠，王文亮，刘斌，刘志华，门可，张景霞，徐剑秋. 胎盘乙型肝炎病毒感染与宫内传播的关系中华妇产科杂志1999，34（7）：392-6
- [李淑红](http://www.cnki.net/kcms/detail/%20%20%20%20%20%20%20%20%20%20%20%20%20%20%20%20/kcms/detail/search.aspx?dbcode=CJFQ&sfield=au&skey=%e6%9d%8e%e6%b7%91%e7%ba%a2&code=23111745;22815202;23109963;23992229;23992230;)； [刘志芬](http://www.cnki.net/kcms/detail/%20%20%20%20%20%20%20%20%20%20%20%20%20%20%20%20/kcms/detail/search.aspx?dbcode=CJFQ&sfield=au&skey=%e5%88%98%e5%bf%97%e8%8a%ac&code=23111745;22815202;23109963;23992229;23992230;)； [贾蓓](http://www.cnki.net/kcms/detail/%20%20%20%20%20%20%20%20%20%20%20%20%20%20%20%20/kcms/detail/search.aspx?dbcode=CJFQ&sfield=au&skey=%e8%b4%be%e8%93%93&code=23111745;22815202;23109963;23992229;23992230;)； [孙红](http://www.cnki.net/kcms/detail/%20%20%20%20%20%20%20%20%20%20%20%20%20%20%20%20/kcms/detail/search.aspx?dbcode=CJFQ&sfield=au&skey=%e5%ad%99%e7%ba%a2&code=23111745;22815202;23109963;23992229;23992230;)； [盖淑坤](http://www.cnki.net/kcms/detail/%20%20%20%20%20%20%20%20%20%20%20%20%20%20%20%20/kcms/detail/search.aspx?dbcode=CJFQ&sfield=au&skey=%e7%9b%96%e6%b7%91%e5%9d%a4&code=23111745;22815202;23109963;23992229;23992230;). HBV感染的CD68细胞在隐匿性HBV感染产妇胎盘中的分布及与宫内感染的关系. 中国优生与遗传杂志，2010, 18（2）：53-4
- [Zhu Q, Lu Q, Gu X, et al. A preliminary study on interruption of HBV transmission in uterus. Chin Med J (Engl) 1997; 110:145.](http://www.uptodate.com/contents/hepatitis-viruses-and-the-newborn-clinical-manifestations-and-treatment/abstract/16)
- [Beasley RP, Hwang LY, Stevens CE, et al. Efficacy of hepatitis B immune globulin for prevention of perinatal transmission of the hepatitis B virus carrier state: final report of a randomized double-blind, placebo-controlled trial. Hepatology 1983; 3:135.](http://www.uptodate.com/contents/hepatitis-viruses-and-the-newborn-clinical-manifestations-and-treatment/abstract/17)
- [Stevens CE, Toy PT, Tong MJ, et al. Perinatal hepatitis B virus transmission in the United States. Prevention by passive-active immunization. JAMA 1985; 253:1740.](http://www.uptodate.com/contents/hepatitis-viruses-and-the-newborn-clinical-manifestations-and-treatment/abstract/18)
- [Schalm SW, Mazel JA, de Gast GC, et al. Prevention of hepatitis B infection in newborns through mass screening and delayed vaccination of all infants of mothers with hepatitis B surface antigen. Pediatrics 1989; 83:1041.](http://www.uptodate.com/contents/hepatitis-viruses-and-the-newborn-clinical-manifestations-and-treatment/abstract/19)
- [Krugman S. Hepatitis B virus and the neonate. Ann N Y Acad Sci 1988; 549:129.](http://www.uptodate.com/contents/hepatitis-viruses-and-the-newborn-clinical-manifestations-and-treatment/abstract/23)
- [Burk RD, Hwang LY, Ho GY, et al. Outcome of perinatal hepatitis B virus exposure is dependent on maternal virus load. J Infect Dis 1994; 170:1418.](http://www.uptodate.com/contents/hepatitis-viruses-and-the-newborn-clinical-manifestations-and-treatment/abstract/24)
- GQ Bai, QH Li,et al. The study on role of peripheral blood mononuclear cell in HBV intrauterine infection. [Arch Gynecol Obstet.](http://www.ncbi.nlm.nih.gov/pubmed/20107823##) 2011Feb;283(2):317-21.
- 乙型肝炎病毒母婴传播预防临床指南2013年 中华医学会妇产科学分会产科学组
- Shi Z, Yang Y, Ma L, et al. Lamivudine in late pregnancy to interrupt in utero transmission of hepatitis B virus: a systematic review and meta-analysis. Obstet Gynecol 2010; 116:147
- [曹敏恺](http://www.cnki.net/kcms/detail/%20%20%20%20%20%20%20%20%20%20%20%20%20%20%20%20/kcms/detail/search.aspx?dbcode=CJFQ&sfield=au&skey=%e6%9b%b9%e6%95%8f%e6%81%ba&code=25053562;22757500;24706277;06614682;23118309;)； [韩国荣](http://www.cnki.net/kcms/detail/%20%20%20%20%20%20%20%20%20%20%20%20%20%20%20%20/kcms/detail/search.aspx?dbcode=CJFQ&sfield=au&skey=%e9%9f%a9%e5%9b%bd%e8%8d%a3&code=25053562;22757500;24706277;06614682;23118309;)； [江红秀](http://www.cnki.net/kcms/detail/%20%20%20%20%20%20%20%20%20%20%20%20%20%20%20%20/kcms/detail/search.aspx?dbcode=CJFQ&sfield=au&skey=%e6%b1%9f%e7%ba%a2%e7%a7%80&code=25053562;22757500;24706277;06614682;23118309;)； [孙梅](http://www.cnki.net/kcms/detail/%20%20%20%20%20%20%20%20%20%20%20%20%20%20%20%20/kcms/detail/search.aspx?dbcode=CJFQ&sfield=au&skey=%e5%ad%99%e6%a2%85&code=25053562;22757500;24706277;06614682;23118309;)； [王翠敏](http://www.cnki.net/kcms/detail/%20%20%20%20%20%20%20%20%20%20%20%20%20%20%20%20/kcms/detail/search.aspx?dbcode=CJFQ&sfield=au&skey=%e7%8e%8b%e7%bf%a0%e6%95%8f&code=25053562;22757500;24706277;06614682;23118309;). HBeAg+HBV DNA高滴度乙肝孕妇替比夫定治疗对胎盘HBV感染的影响. 江苏医药，2011,37（4），419-510
- [André FE, Zuckerman AJ. Review: protective efficacy of hepatitis B vaccines in neonates. J Med Virol 1994; 44:144.](http://www.uptodate.com/contents/hepatitis-viruses-and-the-newborn-clinical-manifestations-and-treatment/abstract/39)
- Committee on Infectious Disease American Academy of Pediatrics. Hepatitis B. In: Red Book: 2012 Report of the Committee on Infectious Diseases, 29th ed, Pickering, LK (Eds), American Academy of Pediatrics, Elk Grove Village, IL 2012. p.369.
- [Lee C, Gong Y, Brok J, et al. Hepatitis B immunisation for newborn infants of hepatitis B surface antigen-positive mothers. Cochrane Database Syst Rev 2006; :CD004790.](http://www.uptodate.com/contents/hepatitis-viruses-and-the-newborn-clinical-manifestations-and-treatment/abstract/40)
- American Academy of Pediatrics. Human milk. In: Red Book: 2012 Report of the Committee on Infectious Diseases, 29th, Pickering LK. (Ed), 2012. p.126.
